# Assessing viability of extracorporeal preserved muscle transplants using external field stimulation: a novel tool to improve methods prolonging bridge-to-transplantation time

**DOI:** 10.1038/srep11956

**Published:** 2015-07-06

**Authors:** Christian D. Taeger, Oliver Friedrich, Adrian Dragu, Annika Weigand, Frieder Hobe, Caroline Drechsler, Carol I. Geppert, Andreas Arkudas, Frank Münch, Rainer Buchholz, Charlotte Pollmann, Axel Schramm, Torsten Birkholz, Raymund E. Horch, Konstantin Präbst

**Affiliations:** 1 Department of Plastic and Hand Surgery, Friedrich-Alexander University Erlangen-Nürnberg; 2 Institute of Medical Biotechnology, Friedrich-Alexander University Erlangen-Nürnberg; 3 Department of Plastic and Hand Surgery, St. Georg Hospital, Leipzig; 4 Institute of Bioprocess Engineering, Friedrich-Alexander University Erlangen-Nürnberg; 5 Chair of Pathology and Anatomy, Friedrich-Alexander University Erlangen-Nürnberg; 6 Department of Paediatric Cardiac Surgery, Friedrich-Alexander University Erlangen-Nürnberg; 7 Department of Neurology, Friedrich-Alexander University Erlangen-Nürnberg; 8 Department of Anesthesiology, Friedrich-Alexander University Erlangen-Nürnberg.

## Abstract

Preventing ischemia-related cell damage is a priority when preserving tissue for transplantation. Perfusion protocols have been established for a variety of applications and proven to be superior to procedures used in clinical routine. Extracorporeal perfusion of muscle tissue though cumbersome is highly desirable since it is highly susceptible to ischemia-related damage. To show the efficacy of different perfusion protocols external field stimulation can be used to immediately visualize improvement or deterioration of the tissue during active and running perfusion protocols. This method has been used to show the superiority of extracorporeal perfusion using porcine *rectus abdominis* muscles perfused with heparinized saline solution. Perfused muscles showed statistically significant higher ability to exert force compared to nonperfused ones. These findings can be confirmed using Annexin V as marker for cell damage, perfusion of muscle tissue limits damage significantly compared to nonperfused tissue. The combination of extracorporeal perfusion and external field stimulation may improve organ conservation research.

Preserving tissue from ischemia-related cell damage is one of the major issues in transplantation and reconstructive medicine. Isolated skeletal muscle tissue is extremely sensitive towards ischemia-related damage^1^,^2^, making muscle tissue an ideal model for research related to ischemia-related cell damage regarding transplantation, revascularisation and replantation. In physical and anatomical studies it could be demonstrated that irreversible muscle cell damage starts after three hours of ischemia and is nearly complete after six hours^3^. Attempts to prolong tissue viability and functionality that have been delevoped and thoroughly tested in the last decade are quite diversified, including cold storage, warm storage, perfusion with blood or blood-like substitutes^4^,^5^,^6^,^7^,^8^,^9^,^10^,^11^,^12^,^13^,^14^. Current standard conservation protocols for organs and amputated extremities are constricted to ischemic cold storage. *Ex vivo* survival of these transplants is still very limited, rendering improved conservation protocols highly desirable. Perfusion protocols are able to elongate *ex vivo* survival in different types of cases, including organ and extremity storage^6^,^9^,^13^,^15^,^16^. Therefore we would like to introduce a novel research tool to improve methods prolonging bridge-to-transplantation time using external field stimulation in an extracorporeal perfusion (EP) setting in combination with porcine *rectus abdominis* muscle.

To assess the efficacy of developed conservation protocols, numerous methods have been developed to evaluate tissue viability, functionality or survival probability. Most are located in the field of histology^4^,^5^,^8^,^11^,^17^ and immunohistochemistry^5^,^9^,^11^,^17^,^18^. The vast majority of these methods demands complex, costly and time-consuming protocols and delivers narrow, localised results from a retrospective point of view. Its impact on global tissue viability and functionality can be estimated only to a certain degree with a low level of safety when transferring these results to clinical daily routine. Moreover, they cannot reflect the functional integrity of transplants, in case of muscle tissue with regard to excitability and force generation capabilities. Thus, we would like to show a method of evaluating the efficacy of tissue conservation protocols assessing muscle functionality using contactless external field stimulation (EFS) via an electrolyte solution to determine muscular viability.

The presented study aims to reveal novel methods to preserve an excised skeletal muscle flap by means of EP, which supplies the muscular tissue with oxygen and other nutrients. To determine potential beneficial effects of extracorporeal perfusion, one muscle flap was used as control, while the other was used in perfusion experiments. The control received only a single flush of 20 ml of heparinized crystalloid fluid to remove intravasal blood residue but no further treatment as it is performed in clinical routine. The other muscle flap was continuously perfused with oxygen saturated heparinized crystalloid fluid with a flow rate of 600 ml/h. The perfusate was reoxygenated via ambient air. Skeletal muscle was chosen because of its high sensitivity to ischemia-related cell damage. This way a better understanding of the ongoing tissue damage during ischemia might be achieved by figuring out how conservation parameters e.g. perfusate compositions, nutrients, oxygen supply, intravascular pressure, temperature and drug treatment in general and to what extent each factor individually contributes to the survival of muscle tissue. Different sets of these parameters and their influence on muscle viability and functionality can be tested with relatively low effort and high level of reliability. This way, therapy adjustments and conservation protocols can be evaluated during ongoing therapy and changes in viability and functionality are accessible immediately. These findings could help to improve current clinical daily routine conservation protocols. Combining EP with EFS creates a research tool which allows to investigate influencing factors and simple modifications of active, ongoing conservation protocols online.

## Results

### Characterization of porcine *rectus abdominis* muscle

**Figure 1:**
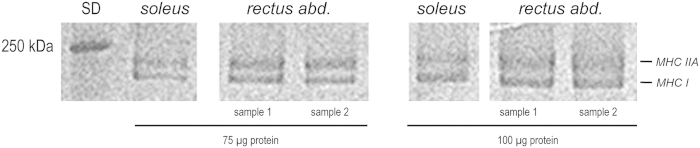
Myosin heavy chain (MHC) isoforms in porcine *rectus abdominis* muscle. Either 75 μg or 100 μg of total protein from two homogenized porcine abdominal wall muscle samples were run on an 8% polyacrylamide SDS gel alongside with a murine *soleus* muscle sample (a pure slow-twitch muscle). Coomassie-stained lanes reveal identical MHC isoform expression patterns in murine *soleus* and porcine *rectus abdominis* muscles, verifying the latter as a slow-twitch muscle.

Since the myosin heavy chain composition of porcine *rectus abdominis* muscle is poorly characterized in the literature, we performed SDS PAGE analysis of porcine *rectus abdominis* muscle homogenate samples. In order to classify the myosin heavy chain (MHC) distribution towards known compositions in a typical slow-twitch muscle, we also ran samples from mouse *soleus* muscle. We chose this internal standard since our previous study in human *rectus abdominis* muscle produced reliable results in classifying human abdominal muscle as a slow-twitch muscle in control patients^19^. Figure 1 shows Coomassie stained lanes from the same gel where 75 μg and 100 μg protein input was separated on an 8% polyacrylamide gel. The two porcine *rectus abdominis* samples produce identical bands as compared to the murine *soleus* sample, i.e. MHC I and MHC IIA^20^, classifying porcine rectus abdominis as a pure slow-twitch muscle.

### Extracorporeal perfusion preserves tissue integrity and reduces apoptotic damage

**Figure 2:**
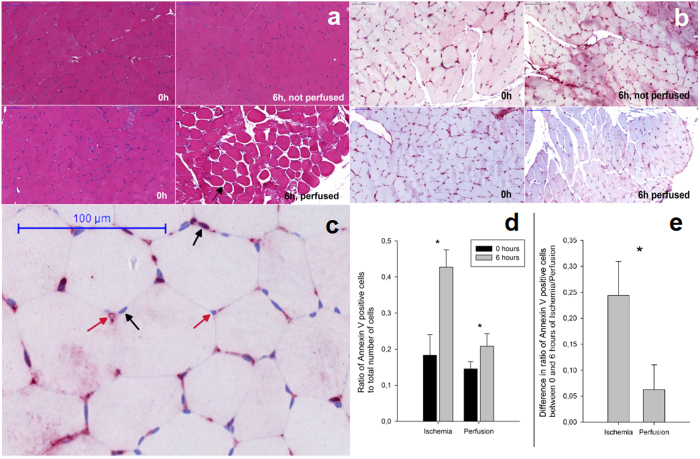
Extracorporeal perfusion preserves tissue integrity and reduces apoptotic damage; (**a)** Histological images of *rectus abdominis* muscle tissue stained with H&E, 200 fold magnification, scale is 100 μm; 6 h, perfused: newly formed intercellular space visible as indicated exemplary by the black arrow, interpreted as interstitial edema; (**b)** Histological images of *rectus abdominis* muscle tissue stained against Annexin V, 200 fold magnification, scale is 100 μm; (**c)** Enlarged histological image of *rectus abdominis* muscle tissue stained with Annexin V, only nuclei within the border of the plasma membrane of skeletal muscle cells are considered myonuclei (as indicated by black arrows). Nuclei close to capillaries as well as outside of the plasma membrane have been excluded in further analysis to reduce unspecific staining and false positives, including endothelial cells and e.g. infiltrating neutrophils (as indicated by red arrows); (**d)** Ratio of Annexin V stained cell nuclei in relation to the total number of cell nuclei; Ratios of positive cell nuclei in relation to total number of cell nuclei for samples taken immediately after harvest (0 hours) before ischemia and after six hours of ischemia (6 hours), immediately after harvest (0 hours) before perfusion and after six hours of perfusion (6 hours). Mean ratios after six hours of ischemia and after six hours of perfusion rise statistically significant (*) compared to their individual values of 0 hours; (**e**) Ratio of Annexin V stained cell nuclei in relation to the total number of cell nuclei; mean difference of ratio changes of individual muscles undergoing six hours of ischemia or perfusion, with a statistically significant (*) lower rise in Annexin V positive cell nuclei of muscles undergoing perfusion. Error bars represent standard deviation of individual values recorded at the same time.

Haemat-oxylin and Eosin (H&E) staining was performed as a means of control (Fig. 2a). It shows significant morphological changes, seen as interstitial and intracellular edema in the muscle particularly in the perfused group after six hours.

No morphological changes between the two samples taken immediately after harvest can be observed (Fig. 2a). After 6 hours of ischemia cellular membranes become more undistinguishable and less cell nuclei are found, indicating a beginning lysis of cell nuclei. After 6 hours of perfusion the cellular integrity is still intact but a severe interstitial and intracellular edema is observable. The formation of an edema can also be seen by evaluation of the muscle’s change in weight after perfusion. After six hours of extracorporeal perfusion, the average weight gain is 99.9% (±22.5%), the muscle almost doubled its initial weight. Without perfusion, there is almost no change in weight after 6 hours 3.0% (±3.1%).

To determine the influence of perfusion on tissue compared to a non-perfused muscle flap, immunohistochemistry was performed using Annexin V as marker for induced damage (Fig. 2b). Therefore, a biopsy was taken at two specific intervals, immediately after harvest of the muscle and after six hours in both groups.

To determine whether the tissues possibly acquired apoptotic damage, the nuclei positive for Annexin V staining were put in relation to the total number of nuclei. Cell clusters in capillaries, for example, have not been included (Fig. 2c). Similar to the H&E stains, the edema formation due to perfusion can also be observed here.

The evaluation of Annexin V (Fig. 2d) shows that even immediately after harvest, up to 18.3% (±5.7%) of total observable cell nuclei were stained positive. After six hours of ischemia, a statistically significant change (p = 0.0001) in Annexin V stained cells occurred, as on average, 42.7% (±4.8%) of the total cell nuclei were positive. The average ratio of positive nuclei of the muscle group before undergoing perfusion was 14.5% (±2.0%), without a statistical significance to the 0 hour value of the group before undergoing ischemia (p = 0.12). This number rises to statistically significant values (p = 0.007) to an average of 20.8% (±3.5%). For statistical analysis, the individual change in the ratio of Annexin V positive nuclei was determined immediately after harvest and six hours of ischemia and perfusion and averaged according to their treatment group (Fig. 2e). Muscles suffering from ischemia showed an average increase in positive nuclei of 24.4% (±6.5%) while undergoing six hours of perfusion, their resulting average rise of 6.3% (±4.8%) is statistically significant (p = 0.001).

### Oxygen consumption during active perfusion

**Figure 3:**
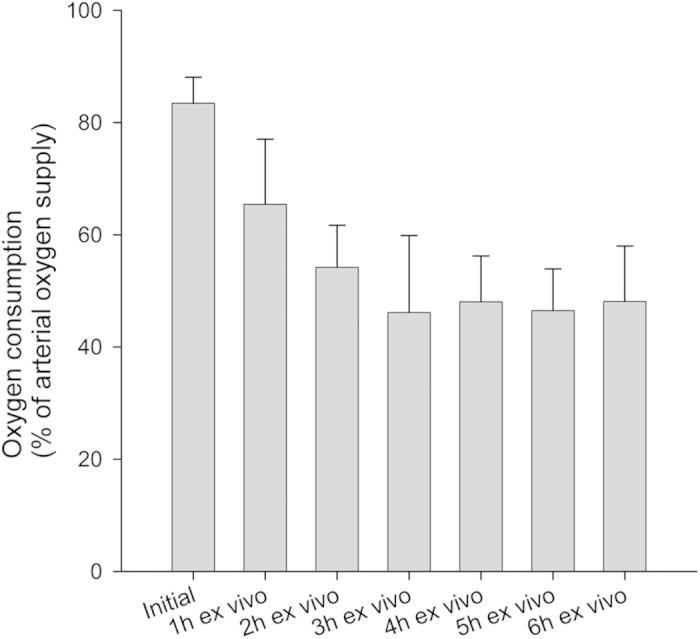
Oxygen consumption of the muscles during active perfusion at different points of time. Oxygen consumption is illustrated in percent of the supplied arterial pO_2_. Error bars represent standard deviation of individual values recorded at the same time.

The oxygen consumption of the perfused muscles was determined continuously during active perfusion. By comparing the oxygen partial pressure (pO_2_) of the perfusate on arterial and venous side, an oxygen consumption of the muscle tissue can be determined (Fig. 3). The pO_2_ on the arterial side was maintained at 100% ambient air saturation by a secondary reoxygenation circuit containing a micro-porous polypropylene membrane.

Initially the perfused tissue consumed 83.4% (±4.6%) of the supplied oxygen. During further perfusion oxygen consumption was reduced over the first 2 hours of the experiment (one hour: 65.4% (±11.6%), two hours: 54.2% (±7.5%)) but reached a stable value between 46.1% (±13.7%) and 48.1% (9.9%) after 3 hours of perfusion. The nonperfused muscle is considered to be suffering from a complete lack of oxygen, as has been shown in previous publications^21^.

### Extracorporeal perfusion helps maintaining muscular response to electrical stimulation

**Figure 4:**
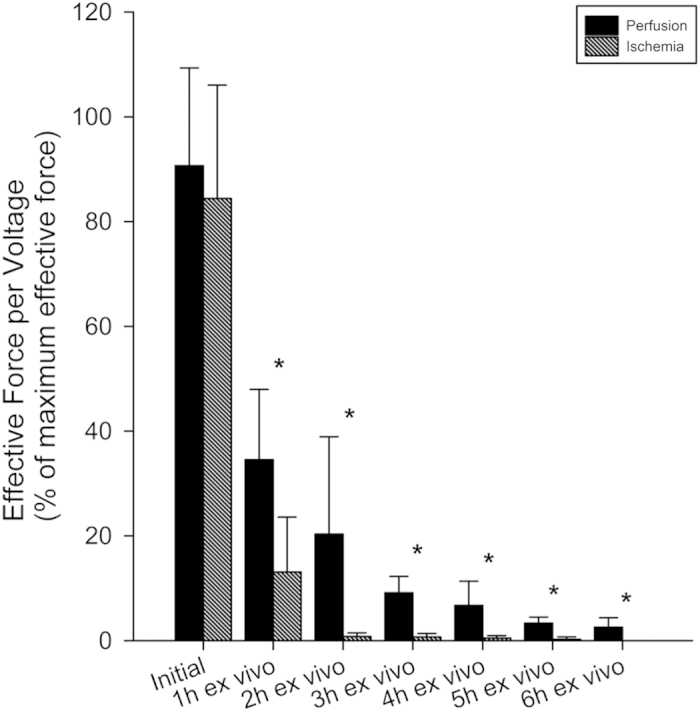
Normalized effective force per voltage of muscles undergoing ischemia or perfusion at different points of time; *indicates a statistically significant difference of effective force exerted by muscles undergoing ischemia and of muscles undergoing perfusion at the same point of time after harvest. Error bars represent standard deviation of individual values recorded at the same time.

In order to relate force decrements from different muscles of different length and cross-section to initial maximum force as a relative measure between muscle flaps, the following normalization procedure proved to be useful: Recorded maximum forces at different points of time are normalized both with reference to the voltage used for stimulation as well as to the maximum value of effective force. The resulting effective force at different points of time is shown in Fig. 4.

In most cases, initial force equals maximum achievable force. In some cases though, the muscle increased its exerted force, which results in a slightly lower initial force. Within one hour of ischemia/perfusion exerted isometric force declined rapidly. The exerted force of the perfused muscles does not exceed 50% of its maximum value, while the highest observable force of the muscle suffering from ischemia does not reach even 25%. With ongoing time of ischemia, the average recorded effective force of unperfused muscles diminishes under a threshold of 1% of maximum effective force, while the number of muscles not reacting to eletrical stimulation increases. After two hours *ex vivo* without perfusion, two out of five flaps did not exert sufficient effective force and were no longer able to react even to higher voltages. After five hours *ex vivo*, only one ischemic flap was still able to exert measurable force but failed to do so after half an hour later. This means that at the end of the preset experimental time, no single ischemic muscle was still able to react to electrical stimulation. Using EP, all muscles conserved *ex vivo* were still able to exert force after the preset experimental time of six hours. The effective force declines extensively at the initation of EP, but with ongoing time, the loss in effective force decreases. Continuous EP extends the time of possible stimulation markedly. All average effective forces of the perfused group were significantly larger than the effective forces within the ischemia group (one hour: p = 0.002, two hours: p = 0.04, three hours: p = 0.001, four hours: p = 0.01, five hours: p = 0.001, six hours: p = 0.01).

## Discussion

Perfusion and oxygenation improve the tissue’s viability and functionality. These results are consistent with our preliminary findings that supplying oxygen while maintaining continuous perfusion reduces the number of apoptotic cells and therefore tissue damage even after one hour *ex vivo* compared to the clinical standard, which is represented by the unperfused control group^22^. Continuous perfusion of muscle transplants can be easily performed and maintained for several hours. One must take into account the formation of edema during active perfusion, though. Edema formation might be reduced by using colloidal perfusion solutions or altering perfusion protocols by reducing flow rates and decreasing pressure thresholds.

Annexin V is able to specifically bind to externalized Phosphatidylserine (PS) of apoptotic cells^23^,^24^ and has been used in various applications for detecting cellular apoptosis^25^,^26^,^27^,^28^ including skeletal muscle^29^,^30^. Wang *et al.* studied the effects of ischemia/perfusion damage on rat *gracilis* muscle and found that apart from necrosis, apoptosis was increased significantly after increased time of ischemia and perfusion^30^. Furthermore, apoptosis in skeletal muscles as a formation of multinucleated cells is quite distinctive from mononuclear cells. Rather than a wholesale cellular decay, individual myonuclei appear to be targeted. The muscle cell can undergo individual myonuclear apoptosis as well as complete cell death, where during ischemia and reperfusion the former appears to be the case^31^,^32^. In our study, incorporated Annexin V^33^ was used to determine cell damage caused by either ischemia or perfusion. We found that continuous extracorporeal perfusion could protect muscle tissue from increased cell damage. This relates at least to the setting of porcine slow-twitch muscle in our study, for which we provide the very first evidence in our MHC profiling. In this context, porcine *rectus abdominis* muscle shows the same slow-twitch MHC I and MHC IIA isoform expression patterns as found in human abdominal muscle from control patients^19^.

Both histological analysis and EFS show that continuous perfusion with a sufficient supply of oxygen of muscle tissue significantly decreases apoptotic responses and increases maintained muscle function. As outlined above, in this study, we performed the extracorporeal perfusion over a time-period of six hours, because (i) this is supposed to be a limiting time regarding ischemia-related cell damage of skeletal muscle and (ii) to evaluate the efficacy of the conservation protocol. It ensures mainly the supply of oxygen during the extracorporeal conservation through the native vessel system instead of simple diffusion through the muscle’s surface, which is shown to be insufficient. The internal tissue oxygen level reaches 0% within minutes of ischemia^21^. Although the oxygen consumption of the perfused muscle tissue decreases during the first two to three hours, a stable value of oxygen consumption can be achieved even after six hours of perfusion. This decrease of oxygen consumption could be explained with a progressing decrease of metabolism due to a growing lack of substances. After six hours, the lack of oxygen supply causes significant changes, seen as an increase in apoptotic cells and maintained functionality between ischemic and perfused muscles. The ratio of apoptotic cells under ischemic tissue conditions rises by ~25%. After the same time, no single ischemic muscle was able to react to electrical stimulation. With the use of EP this can be improved. The ratio of apoptotic cells is increased by only ~6% and all muscles being perfused maintained a certain level of functionality. The lack of decline inducible reaction to EFS can be a sign of cellular damage, without the need of detrimental histological sampling.

Monitoring the muscle force development allows a direct and instant assessment of its viability and functionality. With the approach of a homogenous global stimulation, the entire muscle’s viability can be observed and influences of different treatments can be assessed globally. Therefore, electrical stimulation can be an important tool in ischemia-related transplantation research. According to the data of this study there is a high correlation between preserving viability and functionality of a muscle transplant in the absence of ischemia.

The procedure of EP has already been applied successfully in the field of organ preservation. Extracorporeal perfusion of renal grafts reduces the risk of delayed graft function and improves graft survival compared to static cold storage^10^,^14^. Warnecke *et al.* adapted the method of EP and implemented a device for storing and restoring a human lung^13^. Perfusing the lung’s vascular system while maintaining active ventilation improved the organ’s viability markers. Guarrera *et al.* used EP for the conservation of liver tissue^9^. Though providing promising results and new insights to understand the mechanisms of ischemia-related cell damage, in clinical daily routine, the standard procedure of organ and tissue preservation is still cold storage.

In the field of reconstructive medicine with replantation of extremities or free tissue transplantation, the use of EP did not have any impact on clinical practice, but has been the focus of transplantational research in the last decades^4^,^7^,^8^,^34^. The superiority of perfusion over ischemic cold storage has already been shown for skeletal muscles^35^. Constantinescu *et al.* introduced EP as a tool for preserving whole extremities in a porcine model after prolonged ischemia^5^. Here, also a method was introduced to prove muscle functionality as marker for tissue viability, but without quantification of the muscle’s ability to exert force^5^. Their research focused on the feasibility of extracorporal perfusion on amputated limbs with the use of autologous blood. This was confirmed by Müller *et al.* by replanting blood perfused extremities after prolonged ischemia^11^. Although the approach of using blood in EP of muscle transplants is obvious, it has major drawbacks. First, autologous blood is not available in unlimited supplies. Second, clotting is a permanent threat and the operation costs are immense. Furthermore, the use of whole autologous blood did not prove feasible as it did not show any superiority compared to Jonosteril in a one hour perfusion setting in a previous experimental setting^18^. Based on the results of Constantinescu’s research, a method was introduced perfusing isolated muscle transplants^36^ with alternative perfusate substitutes, including heparinized crystalloid fluid and heparinized cardioplegic solution^22^ with promising results.

Using isolated muscles in contraction experiments has been a standard procedure in animal models and *in vivo* in human patients. Explanting isolated muscles after *in vivo* testing of drug candidates or therapy adjustments allows studying functionality of muscle tissue after completed therapy protocols. Determining muscle function allows evaluation of potential therapeutics for muscle pathology^37^. Using isolated muscles allows a more profound evaluation of effects on muscular tissue. To assess chronological effects of therapeutic interventions, one would have to explant several muscles after defined points in time. To assess effects directly on a cellular or subcellular level, isolated permeabilized muscle fibers or skinned muscle fibre bundles can be tested. By emerging these cells/cell compartments in different solutions containing components of interest, their direct influence can be determined immediately and consecutively^38^,^39^. Mechanisms on a cellular level may not be fully reflected on the whole organ scale though, e.g. due to differential effects on organ areas or biomechanical buffering by extracellular matrix, for instance. Furthermore, using isolated muscle tissue, natural stimulation conduits are not influenced by nerve conditions and other influencing tissue. We thus would like to introduce a method that involves isolated use of whole muscles with direct access to study immediate and temporal effects of different conservation protocols on the functionality of free muscle transplants. This real-time bio-tool allows simple and immediate data acquisition, with which even small modifications of conservation protocols can be evaluated immediately.

Although interesting prospects may also be seen through further advancement of tissue engineering (TE) and regenerative medicine (RM), at this time, TE and RM are not clinically available and hence, not yet applicable to the human patients to replace the need of human organs and tissue^40^,^41^,^42^. Therefore, improving conservation protocols of transplants still remains of utmost importance to deal with the limited resources of organs and transplants.

## Methods

### Animal Model and Surgical Procedure

In this study, n = 5 male mature pigs (Erzeugergemeinschaft Franken Schwaben, Tierische Veredelung, Wertingen-Geratshofen, Germany) were used. Both *rectus abdominis* muscles with the inferior epigastric arteries and veins as pedicle were harvested. The low level of anatomical variation enables uniform operation procedures and the pedicle’s length allows good handling during perfusion^18^,^36^.

Animals were sedated 30 minutes before actual anaesthesia with an intramuscular injection of atropine (Atropinsulfat^®^, Braun, Germany; 0.044 mg/kg) and azaperone (Stresnil^®^, Jansen, Germany; 4 mg/kg). Anaesthesia was started with an intravenous injection of ketamine (Ketanest^®^ 10%, Ceva, Düsseldorf, Deutschland; 15 mg/kg) and pentobarbital (Narcoren^®^, Hallbergmoos, Deutschland; diluted 1:2; 20–40 mg/kg). Subsequently, orotracheal intubation (6.5–7.0 Ch) was performed after topical anaesthesia of the larynx (lidocaine, Xylocain^®^; AstraZeneca, London, UK) under laryngoscopic control. No muscle relaxant was administered. The respirator (Draeger, Luebeck, Germany) was adjusted to controlled artificial respiration with IPPV (intermittent positive pressure ventilation) with weight-adapted respiration volume. Anaesthesia was maintained by inhalation of a gas mixture of 1.5–2% isoflurane (Forene^®^, Abbott GmbH & Co. KG, Wiesbaden, Germany) with air/oxygen. To adjust intraoperative fluid volume loss, the animals received weight-adapted crystalloids (Jonosteril^®^, Fresenius Kabi, Bad Homburg, Germany) during the operation.

After disinfection, a skin incision was made starting at the *xyphoideal processus* continuing to caudal direction. The rectus sheath was opened and the muscle was cut cranially between the third and fourth intersection. The muscle was carefully exposed on its caudal pole following into the lesser pelvis, displaying the inferior epigastric artery and vein. The pedicle was clipped and cut through deep inside the lesser pelvis, and the muscle was removed.

All experiments were approved by the Government of Mittelfranken, Germany No. 65-2532.2-1/10 and the animal care committee of the Friedrich-Alexander-University of Erlangen–Nürnberg. All experiments were carried out in accordance with the relevant guidelines and regulations.

### Perfusion setup

**Figure 5:**
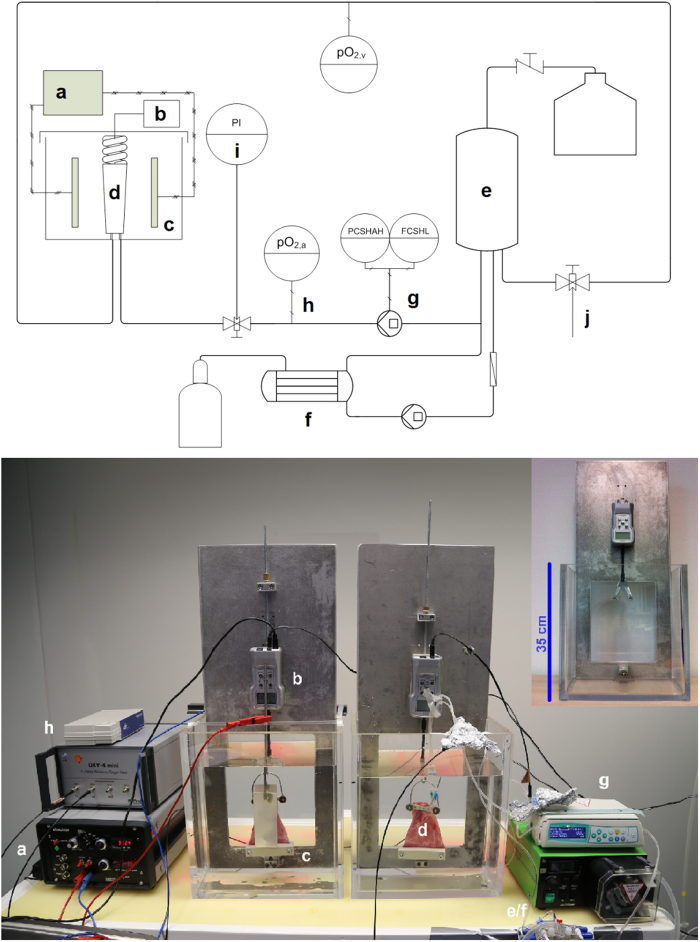
Flow chart of experimental setup; (**a**) Myotronic stimulation unit; (**b**) force gauge; (**c**) electrolyte basin with incorporated silver electrodes; (**d**) musculus rectus abdominis; (**e**) perfusate reservoir; (**f**) oxygenation circuit with hollow fiber membrane oxygenator and gas supply; (**g**) perfusion pump; (**h**) oxygen partial pressure measurement; (**i**) perfusion pressure measurement; (**j**) perfusate sampling; Muscle flap and its suspension to the force gauge as well as the stimulation-unit are located in the bioreactor. The periphery includes measurement devices and the perfusion-system. Perfusate is stored in the reservoir and pumped through a sterile filter and is oxygenated via the hollow fiber membrane perfused with ambient air with a flow rate of 3 l/min to ensure oxygen saturation. Part of the perfusate is brought back into the reservoir and part is diverted into the perfusion circuit connected to the muscles pedicle. On the venous side, the perfusate is extruded and pumped back into the reservoir for reoxygenation. Oxygen partial pressure as well as total perfusion pressure is recorded at the arterial and venous branch of the perfusion circuit. The venous branch also houses a sampling point for perfusate retrieval.

The perfusion setup (Fig. 5) is an extended and advanced version of an earlier stage that has been described previously^21^,^22^,^36^. It consists of a neonatal oxygenator (SAFE Micro^®^, Polystan, Denmark), where one circuit ensures constant perfusion of the tissue flap connected with its pedicle via an arterial and venous anastomosis. A flow and pressure controlled, hermetically sealed peristaltic pump (Infusomat^®^ Space P; Braun Melsungen, Melsungen, Germany) was used as regulating pump for the perfusion circuit. The volumetric flow-rate was adjusted to 600 ml/h as previously tested^36^. The second circuit ensures the oxygenation of the perfusion solution stored in the reservoir bag via a micro-porous polypropylene membrane. The determination of the pO_2_ of the perfusate was performed as previously described^21^,^22^. During active perfusion, pO_2_ of the perfusate were continuously determined on the arterial and venous branch. All parameters were determined using optical sensor systems that allow non-invasive data acquisition with fast response times that allow parameter determination without influences of flow rate and salt concentrations (Sensor type: PSt3 FTC, Transmitter: OXY - 4 - mini, Presens Precision Sensing GmbH). Systolic pressure of the perfusion solution was monitored at the arterial pedicle (SIEMENS Sirecust 961^®^, Siemens AG, Munich, Germany). All experiments were performed at room temperature (20 °C ± 2 °C).

The caudal segment of both *rectus abdominis* muscles (*musculus rectus abdominis sinister/dexter*) was harvested creating two muscle flaps with simple localized blood circulation accessible by one vascular pedicle. To determine potential beneficial effects of extracorporeal perfusion, one muscle flap was used as control, while the other was used in perfusion experiments. The control received only a single flush of 20 ml of heparinized crystalloid fluid to remove intravasal blood residue but no further treatment as it is performed in clinical routine. The other muscle flap was continuously perfused with oxygen saturated heparinized crystalloid fluid with a flow rate of 600 ml/h. The perfusate was reoxygenated via ambient air, while monitoring both arterial and residual venous oxygen partial pressure as well as overall perfusion pressure.

### Characterization of the bioreactor: EFS parameters and potential field distribution maps

Core unit is a bioreactor for suitable storage of free muscle tissue with an integrated electrical field stimulation unit as well as an isometric force gauge to record data of isometric muscle contraction. The gauge can be adjusted in height, which allows optimal fixation of the muscle transplant and alignment of muscle resting tension. The force gauge is based on the principle of a resistance strain gauge (Alluris™ FMI-220B2, Alluris GmbH, Freiburg, Germany). The implemented force gauge has a measurement range of 0–20 N with a measuring frequency of 1000 Hz, an accuracy of 0.2% and a resolution of 0.01 N. Using the muscle’s ability to exert force and comparing the force acquired during the time course of the experiment to its initial force, an estimate for the tissue viability can be deduced. The force and stimulation setup is easily assembled. The muscle is fixated caudally and cranially between a fixation clamp, that also prevents caudal leakage and a transfixing clamp on the cranial side that further prevents muscle tearing on its smaller cranial end, connected to an isometric force gauge. The setting is immersed in a Perspex reactor containing 15 l of electrolyte solution with an average electrical conductibility of 14.1 ± 0.4 mS/cm. On either side of the planar muscle flap, silver plate electrodes (fine silver 999, dimensions: 120 mm × 50 mm) connected to an external stimulation unit were placed in equidistant positions with a total distance of 6 cm between both electrodes. The stimulator (Myotronic™ Stimulator, Myotronic UG, Heidelberg, Germany) generates a monopolar pulsatile electric square waved current. Trains of stimuli can be adjusted as needed. Stimulation frequency can be varied from single pulse up to 150 Hz (with pulse durations shorter than 1 ms) and stimulation voltage can be adjusted between 0 V and 20 V. The frequency of stimuli was set to 100 Hz to reach a fused tetanus of muscle fibres. The operation mode of the stimulator applied in the trials was adjusted to monophasic square waved current. For a frequency of 100 Hz the square waves are seen every 0.01 s with a pulse duration of 1 ms. Bouts of tetani were applied for 10 s.

Using the muscle’s force development as marker for the muscle viability, a uniform stimulation of the entire muscle must be ensured, which is achieved by a homogenous stimulation across the flat surface of the muscle. For this purpose, the field potential inside the stimulation unit was recorded with target stimulation parameters of repeated pulses (pulse duration: 1 ms, pulse frequency: 100 Hz, stimulation output voltage: 0.166 V∙cm^−1^–0.833 V∙cm^−1^).

**Figure 6:**
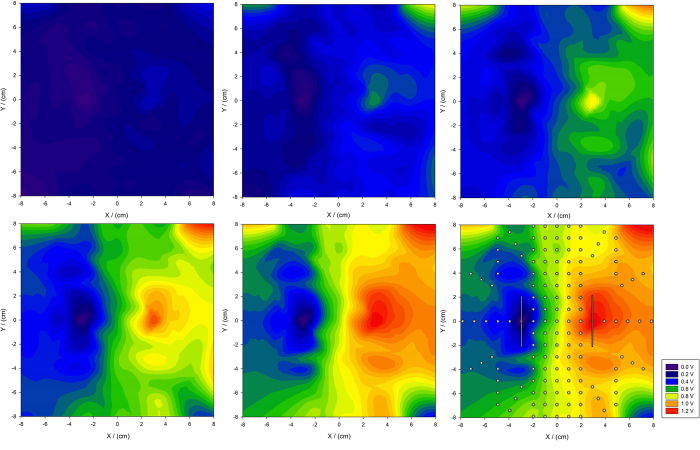
Electrical field distributions at different voltage output settings: (**a**) 0.17 V∙cm^−1^, (**b**) 0.33 V∙cm^−1^, (**c**) 0.50 V∙cm^−1^, (**d**) 0.67 V∙cm^−1^, (**e**) 0.83 V∙cm^−1^, (**f**) 0.83 V∙cm^−1^ with superposed structures: white dots represent measuring point, white bars represent electrode positions.

Figure 6 shows effective voltage maps recorded with one electrode as reference. As stimulation voltage is increased, the effective voltage between both electrodes rises linearly. Furthermore, a homogenous field can be established at the position of the muscle.

### Using muscle tissue to evaluate perfusion to improve conservation compared to diffusion-limited methods

Both muscle flaps, the control flap as well as the perfused one, were placed inside the stimulation setting and pre-stretched with constant force (0.25 N). Stimulation was carried out with the aforementioned pulse parameters. Stimulation pulses were triggered three times in quick succession every 15 minutes with a measurement duration of 10 seconds each and developing force was recorded over a time period of six hours. Muscles were stimulated with an initial stimulation voltage of 0.166 V∙cm^−1^ as sufficient force was generated for determining functionality and overexcessive stimulation may have resulted in inadvertent tissue damage.

### Histological methods

All biopsies were fixed in 4% buffered formalin at ambient temperature and were embedded in paraffin (FFPE). One slide (2–3 μm) per block was taken for immunohistochemistry of Annexin V. H&E stainings were performed according to the protocol of the Leica Autostainer XL (Leica Biosystems Nussloch GmbH, Nussloch, Germany).

### Immunohistochemistry (IHC) of Annexin-V

IHC of Annexin-V was performed as a marker of apoptosis. It may be used to detect translocation of the membrane phospholipid-phosphatidylserine (PS) from the inner to the outer layer of the membrane leaflet by binding specifically PS^43^. PS is usually found on the inner side of the membrane, but during apoptosis, the PS is translocated to the outer membrane. The externalisation of PS from the inner to the outer plasma membrane is a well-established feature of apoptosis^43^,^44^. The staining of Annexin in the plasma membrane, which has also been described in the literature, did not lead to statistically analyzable values. Using Annexin V in cell nuclei on the other hand did, as it accumulates as endogenous protein in the plasmatic coagulation cascade also in damaged cell nuclei, due to the loss of membrane integrity during the late phase of apoptosis^28^,^33^,^45^.

The slides (2–3 μm) were pretreated with a sodium citrate buffer (pH 6,0). After flushing with a tris-buffer a blocking solution (Reagent1) was added for 5 min before incubating with antibodies against Annexin V antibody (ab14196; Abcam, Cambridge, UK) overnight (dilution 1:500). After adding PostBlock (Reagent 2) for 30 min and AP-Polymer (Reagent 3) for another 30 min, the Fast-Red-reaction was added for 15–20 min (room temperature). The section was then counterstained for nucleus detection with haematoxylin before mounting.

After IHC, the slides were digitized using a digital slide scanner (Panoramic-Midi; and Panoramic Flash 250, 3D-Histech AG, Budapest, Hungary). The digital slides were independently analyzed by specialists who documented the five HPFs for quality management. In the HPF all cells were counted (IHC negative and positive ones). The quality of the performed IHC and the evaluated region on each slide were evaluated and analyses were done by two specialists to achieve double-blinded quality controls.

Therefore, five HPFs (magnification of 200, studies area: 600 × 400 μm) were distributed randomly over each slide and the total number of all cells was counted by eye.

For evaluating the slides, positive nuclei were predefined as IHC positive and negative nuclei as IHC negative. The number of positive cells was determined in a 400x magnification and the percentage of positive cells in each slide was calculated. In this way, the percentages of positive cells of each IHC of each slide at 0 hours and six hours after explantation were calculated.

### SDS PAGE of myosin heavy chains (MHC)

In order to separate MHC profiles via electrophoresis, two porcine *rectus abdominis* muscle samples were homogenized. As an internal standard, we also used a mouse *soleus* muscle, a pure slow-twitch muscle predominently expressing MHC I and MHC IIa isoforms^20^. The protocol was already desribed in previous publications^19^,^20^. Briefly, protein was extracted using a muscle lysis buffer (5 mM TRIS, 10% SDS, 0.2 M DTT, 1 mM EDTA, 100 mM NaF, 50 mM ß-Glycerophosphate, 2 mM Na3VO4, 1 mM PMSF supplemented with protease inhibitors; per 10 mg of sample, 200 μl lysis buffer was used). Taring of samples was performed with a Tissue Lyser II (Quiagen, Germany) with 30 shakes per second over 2 min followed by three times two minute centrifugation at 13,000 g. Samples were then heated to 95 °C for 5 min, re-centrifuged and the supernatant added to 8% polyacrylamide SDS gels containing glycerol as described by Talmadge and Roy^46^ after BCA protein concentration determination. Gels were loaded with either 75 μg or 100 μg of protein and run over 24 hrs at 4 °C and 95 V. Gels were then stained with Coomassie staining solution and documented using a Fusion-SL Advanced 4.2 MP system (Vilber Lourmat, Germany). Lanes containing *soleus* and *rectus abdominis* samples were cut from the same gel at the same height for graphical presentation.

### Statistical analysis

A two-sided paired student’s t-test was utilized to detect statistically significant differences. A p-value of <0.05 was considered significant. All analyses were performed using statistical software SPSS for Windows version 21 (SPSS inc., Chicago, USA).

### Solutions

Jonosteril^®^ used as intravasal perfusate has been purchased as sterile solution from Fresenius Kabi. The electrolyte solution was based on Jonosteril^®^. It contained 140 mmol/l Na^+^, 4 mmol/l K^+^, 1.6 mmol/l Ca^2+^, 1.25 mmol/l Mg^2+^, 110 mmol/l Cl^−^ and 37 mmol/l acetate.

## Additional Information

**How to cite this article**: Taeger, C. D. *et al.* Assessing viability of extracorporeal preserved muscle transplants using external field stimulation: a novel tool to improve methods prolonging bridge-to-transplantation time. *Sci. Rep.* **5**, 11956; doi: 10.1038/srep11956 (2015).
